# Acquired Cardiovascular Diseases in Patients with Pulmonary Hypertension Due to Congenital Heart Disease: A Case Report

**DOI:** 10.3390/medicina60020266

**Published:** 2024-02-03

**Authors:** Eglė Ereminienė, Mantvydas Stuoka, Rasa Ordienė, Jurgita Plisienė, Skaidrius Miliauskas, Eglė Tamulėnaitė

**Affiliations:** 1 Department of Cardiology, Medical Academy, Lithuanian University of Health Sciences, LT-44307 Kaunas, Lithuania; mantvydas.stuoka@kaunoklinikos.lt (M.S.); rasa.ordiene@kaunoklinikos.lt (R.O.); jurgita.plisiene@lsmu.lt (J.P.); egle.tamulenaite@kaunoklinikos.lt (E.T.); 2 Laboratory of Clinical Cardiology, Institute of Cardiology, Lithuanian University of Health Sciences, LT-50162 Kaunas, Lithuania; 3 Society of Cardiologists of Kaunas Region, LT-50103, Kaunas, Lithuania; 4 Department of Pulmonology, Medical Academy, Lithuanian University of Health Sciences, LT-44307 Kaunas, Lithuania; skaidrius.miliauskas@lsmu.lt

**Keywords:** adult congenital heart diseases, pulmonary hypertension, acquired heart diseases, prevention and management

## Abstract

*Background:* Advances in the diagnosis and treatment of congenital heart diseases (CHDs) have resulted in improved survival rates for CHD patients. Up to 90% of individuals with mild CHD and 40% with complex CHD now reach the age of 60. Previous studies have indicated an elevated risk of atherosclerotic cardiovascular disease (ASCVD) and associated risk factors, morbidity, and mortality in adults with congenital heart disease (ACHD). However, there were no comprehensive guidelines for the prevention and management of acquired cardiovascular diseases (CVDs) in ACHD populations until recently. *Case presentation:* A 55-year-old man with Eisenmenger syndrome and comorbidities (arterial hypertension, heart failure, dyslipidemia, hyperuricemia, and a history of pulmonary embolism (PE)) presented with progressive breathlessness. The electrocardiogram (ECG) revealed signs of right ventricle (RV) hypertrophy and overload, while echocardiography showed reduced RV function, RV overload, and severe pulmonary hypertension (PH) signs, and preserved left ventricle (LV) function. After ruling out a new PE episode, acute coronary syndrome (ACS) was diagnosed, and percutaneous intervention was performed within 24–48 h of admission. *Conclusions:* This case highlights the importance of increased awareness of acquired heart diseases in patients with pulmonary hypertension due to CHD.

## 1. Introduction

Pulmonary arterial hypertension (PAH) is prevalent among adults with congenital heart disease (CHD), affecting from 4.2 to 28% of CHD patients [1]. PAH associated with CHD is divided into four clinical and hemodynamic profiles: Eisenmenger syndrome, PAH associated with a predominant moderate to-large systemic-to-pulmonary shunt, PAH associated with a small defect, and PAH associated with a repaired defect [1,2]. Eisenmenger syndrome is characterized by the reversal of large unrepaired systemic-to-pulmonary shunt due to pulmonary vasculopathy and increased pulmonary vascular resistance (PVR) [1]. Patients with PAH associated with CHD have better outcomes compared to those with idiopathic PAH, and the survival of patients with Eisenmenger syndrome is superior compared to cases with unrepaired CHD without Eisenmenger syndrome or patients with persistently increased PVR after shunt closure [2,3].

Recent advancements in diagnostic methods, surgical and medical treatment, and follow-up care have led to increased survival rates among CHD patients, with a growing number reaching advanced ages. It is estimated that up to 90% of patients with mild CHD and 40% with complex CHD reach the age of 60 [4]. As more patients with CHD reach advanced ages, there is a shift in mortality from congenital to acquired disease pathways. Previous studies showed that adults with congenital heart disease (ACHD) have an increased risk of premature atherosclerotic cardiovascular disease (ASCVD), with more complex treatment and worse outcomes due to existing congenital heart defects [5].

Previous European Society of Cardiology (ESC) guidelines (2020 ESC guidelines for the management of ACHD and 2021 ESC guidelines for cardiovascular disease prevention) provided limited information about risk factors and the management of acquired heart diseases in the aging ACHD population. Due to increasing CVD morbidity in advanced-age ACHD patients, a new 2023 ESC clinical consensus statement has been released with the aim of preventing ASCVD and reducing the CVD burden in this expanding ACHD population.

## 2. Clinical Presentation

A 55-year-old patient has been under the care of pulmonary hypertension (PH) center for inoperable CHD and Eisenmenger syndrome. Treatment with specific PH medications, including PDE-5 inhibitors was initiated in 2015. The patient was diagnosed with a congenital heart defect—aortopulmonary window—at the age of 10, and surgical treatment was already contraindicated due to severe PAH with a mean pulmonary artery pressure of 92 mmHg, a PVR of 16 Wood units, and a pulmonary capillary wedge pressure of 11 mmHg.

In 2015, the patient underwent surgical treatment for a brain abscess at a neurosurgery clinic. The patient’s status included persistent left limb weakness and difficulty walking. At the neurorehabilitation clinic, he was diagnosed with v. poplitea thrombosis, complicated by pulmonary embolism (PE). Treatment with anticoagulants was initiated.

Additionally, the patient was diagnosed with grade 2 arterial hypertension, congestive heart failure class III (NYHA), and hyperuricemia. The patient had a normosthenic body type with a BMI of 22.9 kg/m^2^.

At a recent visit to our PH center, the patient complained of increased dyspnea with minimal physical exertion, symptoms had worsened over the past two weeks. No chest pain symptoms were reported during physical activity. On physical examination, the patient had a heart rate of 70 bpm, blood pressure of 146/79 mmHg, no murmurs, and a normal sinus rhythm. Lung auscultation showed no rales, but there was cyanosis and decreased saturation (SpO_2_ 88%). Left leg edema was also observed.

**Figure 1:**
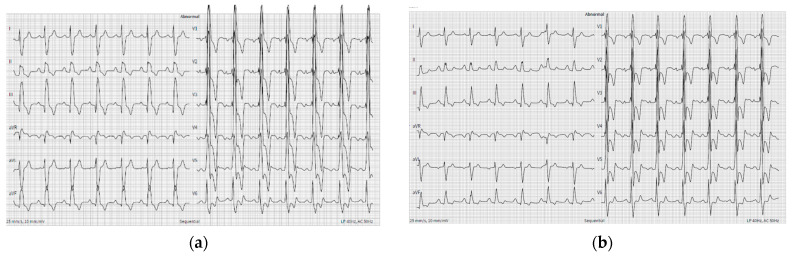
ECG showing right ventricle hypertrophy and RV overload signs ((**a**)—ECG 2 months before recent visit and (**b**)—ECG at recent visit).

**Figure 2:**
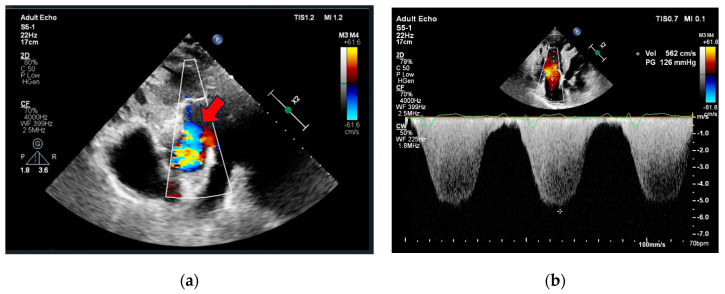
Echocardiography (**a**) parasternal short axis view showing aortopulmonic window (red arrow), (**b**) apical 4-chamber view showing right ventricle dilatation, hypertrophy, severe tricuspid regurgitation, and pulmonary hypertension.

Initial investigations included an electrocardiogram (ECG), laboratory tests (biochemistry, D-dimer, Troponine I and B-Type Natriuretic Peptide levels), chest X-ray, and echocardiography. The case was discussed with pulmonologists at the PH center. Due to the deteriorating condition of the patient (increased dyspnea, elevated BNP levels, RV overload signs in ECG with no dynamic ST-segment and T-wave changes compared to previous ECG, reduced RV function, RV overload, and severe PH signs, preserved LV function without regional wall contraction abnormalities in echocardiography), a new PE episode could not be excluded. Initial clinical data are provided in Table 1 and Figure 1 and Figure 2. The patient was subsequently hospitalized in the pulmonology clinic for further investigation and treatment correction. 

**Figure 3:**
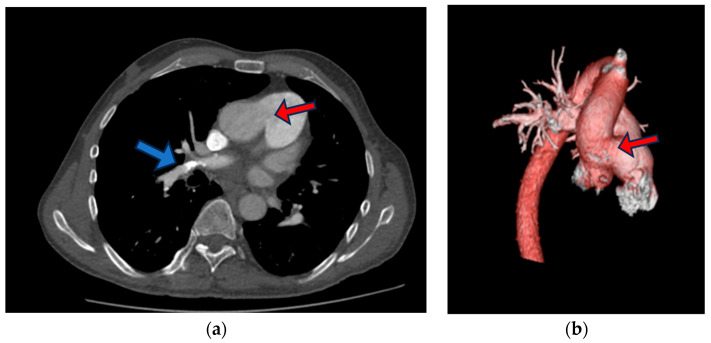
Chest CT (**a**) slide that shows aortopulmonic window (red arrow), right upper lobe anterior segment sub-segmental branches with small filling defects with adjacent calcification (blue arrow), (**b**) three-dimensional (3D) reconstruction of contrast-enhanced CT angiography images of the thoracic aorta and pulmonary artery trunk showing aortopulmonic window (red arrow).

At the pulmonology clinic, an urgent chest computed tomography angiography CTA was performed following the PE protocol. The results revealed insufficient data for an acute PE, showing only signs of pulmonary hypertension, including RV dilatation, RV hypertrophy, and a dilated pulmonary trunk (diameter 37 mm). However, in several right upper lobe anterior segment sub-segmental branches, small filling defects with adjacent calcification were identified—indicative of chronic PE. The size of the defect between the aorta and the proximal part of the pulmonary trunk measured up to ~3.2 cm (Figure 3).

**Figure 4:**
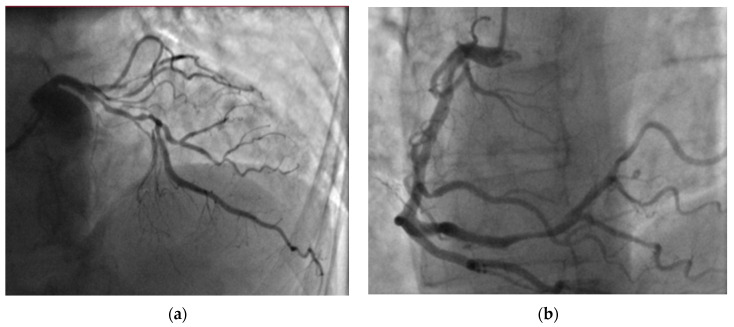
Coronary angiography revealed two-vessel coronary artery disease: (**a**) significant left anterior descending artery stenoses and (**b**) right coronary artery distal stenoses.

Following the exclusion of PE, coronary artery angiography (CA) was performed to investigate the possibility of external compression of the left coronary ostium due to a dilated pulmonary trunk. However, CA revealed a two-vessel CAD with the following CA stenoses: S3—75, S4—100, S6—75, S7—75, S9—90%) (Figure 4). After receiving a loading dose of two antiplatelet agents, the patient underwent a successful percutaneous coronary intervention (PCI) on segments S6, S7, and S8 with 2 drug-eluting stents (40- and 24-mm length).

The final diagnosis was myocardial infarction (MI) without ST elevation, characterized by progressive dyspnea as an anginal equivalent, dynamic TnI level ranging from 0.16 to 0.25 μg/L, and significant CAD observed on CA. Additional laboratory tests revealed untreated hypercholesterolemia (low-density lipoproteins (LDL)—3.88 mmol/L).

The recommended treatment plan included triple antithrombotic therapy for one week after PCI (aspirin, clopidogrel, warfarin), followed by dual therapy for one year (clopidogrel and warfarin). Subsequently, the patient would continue with anticoagulant monotherapy, along with ongoing outpatient medical treatment for PH (PDE-5 inhibitors), AH, and HF using beta-blocker, angiotensin-converting enzyme (ACE) inhibitor, aldosterone receptor antagonist, as well as treatment for hyperuricemia with allopurinol. Statins were added for the management of hypercholesterolemia. In the initial days of hospitalization, oxygen therapy with a nasal oxygen cannula was administered for hypoxia treatment. Once a patient was stabilized, he was referred to cardiac rehabilitation for post-acute MI care.

## 3. Discussion

Acquired heart diseases are a leading cause of death not only in the general population but also in the growing ACHD population. Due to the lack of CVD prevention and management recommendations in ACHD patients, there is a need for collaborative efforts between ACHD specialists and general practitioners to develop effective strategies aimed at reducing the growing cardiovascular burden in this population.

ACHD patients with specific CHDs, such as congenital coronary artery anomalies, the transposition of great arteries post-arterial switch repair or Ross procedures, the coarctation of aorta, and the external compression of the left coronary ostium (due to dilated pulmonary trunk in patients with Eisenmenger syndrome) have significantly increased risks of coronary artery disease (CAD). Due to the high prevalence of abnormal electrocardiograms (ECGs) with conduction abnormalities, ventricular hypertrophy, and overload signs in ACHD patients, it is crucial to carefully assess changes from baseline when evaluating ECG signs of ischemia [5].

Previously, it was believed that patients with Eisenmenger syndrome had reduced risks of ASCVD due to hypoxemia, upregulated nitric oxide, low platelet count, hypocholesterolaemia, and hyperbilirubinemia. However, the improved survival of cyanotic PH patients to advanced age has increased the prevalence of metabolic syndrome or other traditional CVD risk factors, including obesity, hypertension, and diabetes mellitus. Recent data show that the risk of CAD in cyanotic patients is similar to the general population, despite the previously mentioned protective pathophysiological mechanisms. In severe PH cases, a dilated pulmonary trunk may externally compress the left coronary ostium between the pulmonary trunk and aorta, causing angina, dyspnea, or even sudden death [5].

Previous studies revealed that ACHD patients have more ASCVD risk factors in comparison to the general population. Agarwal A. et al.’s study included more than 40,000 adults with CHD, and they evaluated three categories of comorbidities in this population: those commonly associated with CHD (congestive heart failure (HF), pulmonary circulation disorders, and arrhythmias,), and other cardiovascular (hypertension, hypercholesterolemia, CAD, peripheral vascular disease, and stroke) and non-cardiovascular (such as diabetes mellitus, obesity, renal disease, psychiatric disorders, etc.) conditions. The study results showed that CHD patients had a 9.41-, 1.73-, and 1.47-fold higher risk for comorbidities common in CHD, and other cardiovascular and non-cardiovascular comorbidities, respectively. Eisenmenger syndrome patients had the highest comorbidity rate at 65.4%, with 22% diagnosed with arterial hypertension, 8.92% with hyperlipidemia, and 6.49% with CAD [6].

Adults with CHD have a higher burden of adverse cardiovascular events, even in the cases of low complexity CHD, compared to the general population. Saha P. and co-authors compared adverse cardiovascular events, incorporating further statistical analysis for risk estimation unaccounted by conventional cardiovascular risk factors. In a cohort of 2006 individuals with lower-complexity ACHD and 497 983 unexposed individuals, the study revealed that, even after adjustment for conventional risk factors, ACHD patients had a higher risk of adverse cardiovascular events compared to controls. This ranged from a 2-fold increased risk of acute coronary syndrome to a 13-fold increased risk of HF) [7]. These interesting results highlight the complexity of ASCVD mechanisms in the ACHD population, emphasizing the necessity for further investigation. One of the mechanisms leading to increased CVD burden in ACHD patients is the concept of inflamm-aging. Congenital heart defects directly change cardiac hemodynamics, while surgical repair is associated with reperfusion injuries, and prosthetic materials lead to inflammasomes activation [8,9]. Chronic inflammation increases the risk of atherosclerosis progression and insulin resistance, along with tissue-wasting mechanisms that accelerate ASCVD and cachexia [10,11].

Several studies have demonstrated increased morbidity and mortality from ASCVD following cardiovascular events in the ACHD population. Olsen M. and co-authors’ population-based study revealed an increased incidence of MI and 30-day mortality after MI in the CHD population compared to the general population. The study indicated that by the age of 70 years, even 10% of CHD patients would have MI [12]. Olsen M. et al.’s study not only revealed an increased risk of MI but also a higher long-term risk of composite events (recurrent MI, new-onset HF, or CVD death). Even after adjusting for traditional CVD risk factors, the risk of MI remained higher compared to controls, emphasizing the significance of ACHD-related risk factors [13].

The recent ESC clinical consensus statement on acquired cardiovascular diseases in adults with congenital heart disease provides detailed risk stratification and risk factors management plans in the ACHD population [5]. The use of established ESC CVD prevention risk scores, such as Systemic Coronary Risk Estimation 2 (SCORE2) and Systemic Coronary Risk Estimation 2-Older Persons (SCORE2-OP), is recommended in the absence of validated tailored screening tools for ACHD patients. Additionally, the statement outlines specific CHD-related acquired heart disease risk factors. For instance, patients with shunt lesions have an increased risk for paradoxical embolism potentially leading to stroke or embolic MI, and cyanotic lesions have an increased risk of diabetes, arrhythmia, etc. [5].

The ASCVD risk factor management plan comprises two main priorities: firstly, to educate patients and increase their awareness about acquired heart diseases and, secondly, to educate ACHD specialists and general practitioners on how to assess and manage CVD risk factors. Patients should be actively educated about physical activity, a healthy diet, common CVD risk factors, their management, and the importance of medical treatment adherence, as well as the management of psychological stress [5]. Historically, ACHD patients were advised to avoid physical activity. However, according to the newest ESC guidelines on sport and exercise, ACHD patients are advised to engage in regular moderate exercise (60–75% of achieved maximal heart rate during cardiopulmonary exercise test, 30 min 4–5 times per week) [14]. Clinicians should screen for overweight/obesity, repeat basic laboratory tests, recommend oral hygiene, calculate SCORE2 (<70 years)/SCORE2-OP (≥70 years) risks, and advise vaccination (influenza, pneumococcal, COVID-19) every year. Additionally, clinicians should consider exercise/cardiopulmonary exercise tests on a 2-year basis. If symptoms, blood pressure, or ECG changes are provoked, further investigation should be performed. Specialists should also screen for diabetes and perform thyroid function tests on a 3-year basis. Moreover, the recent consensus statement provides preventive measures for specific risk conditions in the ACHD population (HF, arrhythmias, CAD, stroke and systemic embolism, infective endocarditis, frailty) [5]. As previously mentioned, inflamm-aging processes lead to accelerated senescence in ACHD patients, yet there is no specific therapy; but, the guidelines provide some beneficial recommendations, including advice on regular physical activity, a healthy diet, and vitamin D supplements. Pre-clinical research shows the potential benefits of renin-angiotensin-aldosterone system inhibitors, and sodium glucose co-transporter 2 (SGLT2) inhibitors [5].

The present case demonstrated an ACS event in an adult with Eisenmenger syndrome, and multiple comorbidities (arterial hypertension, heart failure, previously untreated dyslipidemia, previous PE, and hyperuricemia). Symptoms were atypical (increased dyspnea), and the ECG was not informative due to RV hypertrophy and overload signs without dynamic ST-segment and T-wave changes. Echocardiography showed severe PH, RV hypertrophy, and RV overload with preserved LV function without regional contraction abnormalities. Thus, the preliminary diagnosis was a new episode of PE. After excluding preliminary diagnosis, a multidisciplinary team investigated external compression of the left coronary ostium due to a dilated pulmonary trunk, but CA revealed a two-vessel CAD. Due to a slightly delayed diagnosis of ACS, PCI was performed 24–48 h after the admission, emphasizing the need for awareness of acquired heart diseases in ACHD patients. Medical CVD therapy was optimized, and the patient was referred to a cardiac rehabilitation clinic.

## 4. Conclusions

Recent advancements in diagnostic and treatment modalities have led to a growing population of patients with PH due to CHD reaching advanced age. There is an urgent need for clinicians to enhance their awareness of prevention and management measures for acquired heart diseases in the ACHD population, as they have increased CVD morbidity and mortality. Diagnosing ACS in patients with PH associated with CHD can be challenging due to abnormal baseline electrocardiogram, and the presence of severe ventricular overload, hypertrophy, and signs of PH in echocardiography.

## Figures and Tables

**Table 1:** Clinical data.

Research	Description
Laboratory blood test	Creatinine 91 µmol/L, Potassium 3.8 mmol/L WBC 4 × 10^9^/L, HGB 214 g/L, PLT 117 × 10^9^/L, BNP 712.3 (0–26.5 ng/L), TnI 0.16 (0–0.04 μg/L), D-dimer 0.1 (0–0.5 mg/L).
ECG	Sinus rhythm, heart rate 59 bpm, RBBB, RVH, and overload signs (Figure 1).
2D TTE	LVEDD 46 mm (LVEDDi 24.08 mm/m^2^), LV EF 50%. RV 48 mm, RA 50 mm, RV wall thickness 10 mm, S’ 6.5 cm/s, FAC 19.1%, moderate TR, PASP 133 mmHg, PA diameter 40 mm (Figure 2).
Chest X-ray	No fluid in the pleural cavities, no infiltration, and congestive signs in the lungs.

WBC—white blood cells, HGB—hemoglobin, PLT—platelet, ECG—electrocardiogram, RBBB—right bundle branch block, RVH—right ventricle hypertrophy, 2D TTE—two-dimensional transthoracic echocardiography, LVEDD—left ventricle end diastolic diameter, LV EF—left ventricle ejection fraction, RA—right atrium, FAC—fractional area change, TR—tricuspid regurgitation, PASP—pulmonary artery systolic pressure, PA—pulmonary artery.

## Data Availability

Not applicable.
